# IGF-1 in autosomal dominant cerebellar ataxia - open-label trial

**DOI:** 10.1186/s40673-014-0013-8

**Published:** 2014-10-02

**Authors:** Irene Sanz-Gallego, Francisco J Rodriguez-de-Rivera, Irene Pulido, Ignacio Torres-Aleman, Javier Arpa

**Affiliations:** Reference Unit of Hereditary Ataxias and Paraplegias, Department of Neurology, IdiPAZ, Hospital Universitario La Paz, Paseo de la Castellana, 261, 28046 Madrid, Spain; Neuroendocrinology Laboratory, Functional and Systems Neurobiology Department, Cajal Institute, CSIC, and CIBERNED, Avda Dr. Arce, 37, 28002 Madrid, Spain

**Keywords:** ADCA, IGF-1 therapy

## Abstract

**Background:**

The objective of this clinical open-label trial was to test the safety, tolerability and efficacy of IGF-1 therapy for autosomal dominant cerebellar ataxia (ADCA) patients.

**Results:**

A total of 19 molecularly confirmed patients with SCA3, 1 patient with SCA6 and 6 patients with SCA7 completed our study. They were 8 females and 18 males, 28 to 74 years of age (average ± SD: 49.3 ± 14.1). Patients were treated with IGF-1 therapy with a dosage of 50 μg/kg twice a day for 12 months. The efficacy of this therapy was assessed by change from baseline on the scale for the assessment and rating of ataxia (SARA). Ten patients, consecutively selected, continued their assigned dosages in a second year open-label extension trial. A statistically significant improvement in SARA scores was observed for patients with SCA3, patients with SCA7 and all patients grouped together after the first year of IGF-1 therapy, while a stabilization of the disease was confirmed during the second year (extension study). The single patient with SCA6 showed 3 improvement points in SARA score after 3 four-month periods of IGF-1 therapy when compared with baseline measurements. Our data indicate that IGF-1 is safe and well tolerated in general.

**Conclusions:**

Our data, in comparison with results from previous cohorts, indicate a trend for IGF-1 treatment to stabilize the disease progression for patients with SCA, indicating that IGF-1 therapy is able to decrease the progressivity of ADCA.

## Background

The discovery of nerve growth factor by Levi-Montalcini and Cohen in 1956 [1] strongly supported the concept that secreted molecules produced by a developing neuron are required for it to survive programmed cell death (apoptosis). Apoptosis is high during embryonic development, during which roughly half of the developing post-mitotic neurons die.

Neurotrophic factors rescue neurons from apoptosis by blocking cell death programs in some physiological contexts. However, neurotrophic factor protection is sometimes limited by pharmacokinetic constraints in well − defined diseases (such as Alzheimer disease and Parkinson’s disease) [2,3].

The insulin-like growth factor system plays important metabolic, trophic and modulatory functions in the central nervous system, increasing cell proliferation, survival and anti-apoptotic responses [4–6]. Central and peripheral insulin-like peptides including insulin, insulin-like growth factor 1 (IGF-1), and IGF-2 exert many effects on the brain cells. They are abundant circulating neuroprotective hormones that might be involved with the control of energy allocation [7]. Disrupted IGF-1 neuroprotective signalling might therefore constitute a common stage in the pathological cascade associated with neuronal death. Second to disease-specific mutations, dysregulation of the IGF-1 signalling pathway is a recurrent finding in mouse models for cerebellar ataxia. The IGF-1 signalling pathway thus represents a common pathological cascade for neuronal cell death that might be a potential therapeutic target. IGF-1 therapy seems to be beneficial for different brain diseases, including various types of cerebellar ataxia in animal models [8–12] and human patients [13]. Whereas the general protective actions of IGF-1 in the brain are well documented, the disease-specific actions of IGF-1, if any, are not yet known. There are several neurodegenerative conditions with probable IGF-1 dysfunction [14]. For instance, two spinocerebellar ataxia (SCA) mouse models (for SCA1 and SCA7) showed a down-regulation of insulin-like growth factor binding protein 5 (*Igfbp5*) transcripts [15]. Two other ataxic diseases with a different aetiology and pathology, ataxia-telangiectasia and Friedreich’s ataxia (FRDA), might also show disturbed IGF-1 function [14]. Shahrabani-Gargir *et al.* [16] showed a significant reduction in basal IGF-1 receptor (IGF-1R) values, together with an impaired IGF-1R response after DNA damage in cells with deficient ataxia-telangiectasia mutated (ATM) function [16]. FRDA shows excess oxidative stress that provokes genotoxic DNA damage [17]. IGF-1 exerts cell-context neuroprotection for frataxin deficiency that might be therapeutically effective in FRDA. Both types of ataxia show cumulative DNA damage [14].

The Insulin/IGF-1 signaling pathway contributes to cell survival and glucagon like peptide-1(GLP-1) has similar functions and growth like properties as insulin/IGF-1. Dysfunction of these pathways seems to contribute to the progressive loss of neurons in Alzheimer’s disease and Parkinson’s disease. These findings have led to numerous studies in preclinical models of neurodegenerative disorders with currently available anti-diabetics [18]. IGF-1 via activation of the serine/threonine kinase Akt/PKB is able to inhibit neuronal death specifically induced by mutant huntingtin containing an expanded polyglutamine (polyQ) stretch [19]. In transgenic animal models of polyQ disorders, there was also evidence of the involvement of insulin/IGF-1 system (IIS) signalling components. IIS proteins were implicated in the modulation of mutant proteins as well as in the disease phenotype [20,21]. Abnormalities in the IIS signalling pathway are also thought to play a part in the physiopathological processes of various neurodegenerative disorders, including Alzheimer’s disease, SCAs and Huntington disease [4,5,22,23].

Our previous small sample clinical trial showed that a subcutaneous dosage of human recombinant IGF-1 of 50 μg/kg twice a day stabilized SCA3 and SCA7 disease progression during the study period [13]. In this paper we added to the previous series the patients of a second study, with the purpose of evaluating a possible increase of the statistical power.

These findings led to make up of the open-label study described here. The primary aim of this study was to demonstrate the safety and tolerability of IGF-1 therapy for patients with autosomal dominant cerebellar ataxia (ADCA). The secondary objective was to evaluate the efficacy of IGF-1 therapy for the treatment of ADCA.

## Results

### Patients

A total of 30 patients, 28 to 74 years of age (average ± SD: 49.3 ± 14.1), were included for this study in the beginning. There were 20 molecularly confirmed patients with SCA3, 2 patient with SCA6 and 8 patients with SCA7. Patients had a baseline SARA score [24] between 8 and 26.75 (average ± SD: 13.2 ± 5.4) (Table 1). Patients were treated with IGF-1 (mecasermin, Increlex®; Ipsen-Pharma) therapy with a dosage of 50 μg/kg twice a day for 12 months. The efficacy of this therapy was assessed by change from baseline on the scale for the assessment and rating of ataxia (SARA) and SF-36v2 Health Survey [25] (see “Methods” session below). Ten patients, consecutively selected, continued their assigned dosages in a second year open-label extension trial.

**Table 1:** **Demographic and clinical variables of the 26 study patients**

**Patients(N)**	**Age (average ± SD) (rank)**	**Gender (M/F,%) (N)**	**Age at onset (average ± SD) (rank)**	**Disease duration (years) (average ± SD) (rank)**	**Expanded CAG repeats (average ± SD) (rank)**	**Baseline SARA (average ± SD) (rank)**
**SCA3 (19)**	**50.32 ± 13.60 (28 – 74)**	**68.4/31.6 (13/6)**	**40.53 ± 12.19 (20 – 62)**	**9.84 ± 7.41 (2 – 30)**	**47.3 ± 33.5 (52 – 75)**	**11.89 ± 4.82 (8 – 28)**
**SCA7 (6)**	**45.71 ± 16.86 (28 – 66)**	**71.4/28.6 (5/2)**	**34.71 ± 10.87 (23 – 48)**	**11.00 ± 6.02 (4 – 18)**	**43.3 ± 24.3 (34 – 50)**	**16.19 ± 6.32 (8–27)**
**SCA6 (1)**	**55**	**100/0**	**48**	**7**	**23**	**14**
**Total (26)**	**49.30 ± 14.12 (28–74)**	**70.37/29.63 (19/8)**	**39.3 ± 11.83 (20 – 62)**	**10.04 ± 6.92 (2 – 30)**	**─**	**13.17 ± 5.38 (8 – 28)**

*N* number.

### Safety and tolerability

In general, IGF-1 was well tolerated by the patients with ADCA. Vital signs showed no remarkable changes from baseline. A total of 4 patients were unable to complete the study: 1 patient with SCA7 died during the follow-up because of aspiration pneumonia; 1 patient with SCA3 withdrew voluntarily; 1 patient with SCA7 was withdrawn because of consecutive fracture and fasting hypoglycaemia; and 1 patient with SCA 6 withdrew because a traffic accident. Of the remaining patients, 2 presented common warts, 2 women gradually changed from straight hair to curly hair and 1 woman showed an alteration in the frequency and duration of her menstrual cycle.

### Efficacy

**Figure 1:**
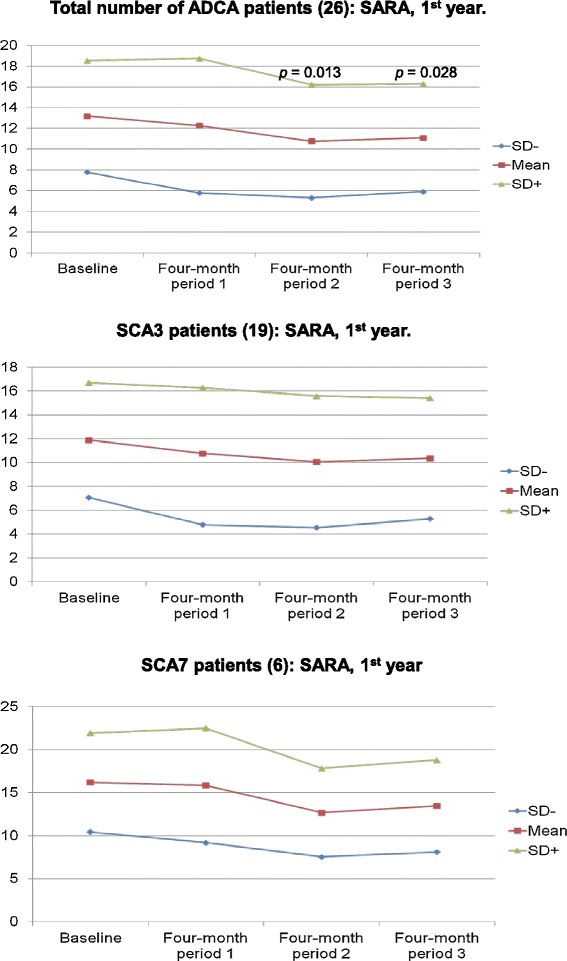
**These charts with mean and standard deviation lines show four-monthly score changes from baseline on the scale for the assessment and rating of ataxia (SARA) during the first year of treatment with IGF-1.** A significant improvement in SARA scores was observed for all patients (26) (top), and the SCA3 (19 patients) (middle), and SCA7 (6 patients) (bottom) subgroups after the first year of IGF-1 treatment.

26 patients, 8 females and 18 males, completed the study. A significant improvement in SARA scores was observed for all patients and the SCA3 and SCA7 subgroups after the first year of IGF-1 treatment (Figure 1). On the Wilcoxon signed ranks test, the SARA score was significantly lower after 1 year of IGF-1 treatment when compared with baseline (p < 0.05). The single patient with SCA6 showed 3 improvement points in SARA score after 3 four-month periods of IGF-1 therapy when compared with baseline measurements. The parameters of SARA that more favorably have been influenced (% score reduction average) by the IGF-1 therapy are nose-finger test (−7.75%), heel-chin slide (−7.5%), stance (−5.8%), gait (−4.25%), and finger chase (−3.25%). The parameters that worse respond are fast alternating hand movements (−0.32%), sitting (+0.5%), and speech disturbance (+1.25%).

The annual worsening rate was estimated for this series to be a SARA score of average ± SD: −1.68 ± 2.8 (95% CI: −2.77 to −0.59), whereas the annual worsening rate for the Jacobi *et al.* SCA3 cohort [26] was estimated to be a SARA score of average ± SD: 1.61 ± 0.12 (95% CI: 1.59 to 1.63; Table 2), and SCA7 SARA score of average ± SD: 1.5 ± 0.9 (95% CI: 0.79 to 2.21) (Table 2). Confidence interval calculated for the measure of treatment effect is not included within the limits of upper and lower bounds of the CI control, which would seem to indicate a decrease in the progression of the disease with IGF-1 therapy. The comparison of our results with those of Jacobi *et al.* in SCA3 and SCA6 [18]*,* and our own SCA7 SARA score, indicated that our patients with ADCA who receive IGF-1 treatment showed a stabilization of disease progression.

**Table 2:** **Annual worsening rate estimated for this series**

**Patients**		**N**	**Annual worsening index (average ± SD)**	**95% confidence interval for mean**	
**1 year − baseline**	**SCA3**	19	−1.54 ± 3.0	−2.89	−0.19
	**SCA7**	6	−2.12 ± 2.1	−3.80	−0.44
	**SCA6**	1	−3	−3	−3
**2** ^**nd**^ **year (extension)**	**SCA3**	6	−0.08 ± 1.11	−0.97	0.81
	**SCA7**	4	0.63 ± 1.93	−1.26	2.52
**Control SCA3**		**139**	1.61 ± 0.12	1.59	1.63
**Control SCA7**		**7**	1.5 ± 0.9	0.79	2.21
**Control SCA6**		**107**	0.35 ± 0.34	0.29	0.41

Comprehensive quantitative account of disease progression was given by Jacobi *et al.* [26]. *N* number.

**Figure 2:**
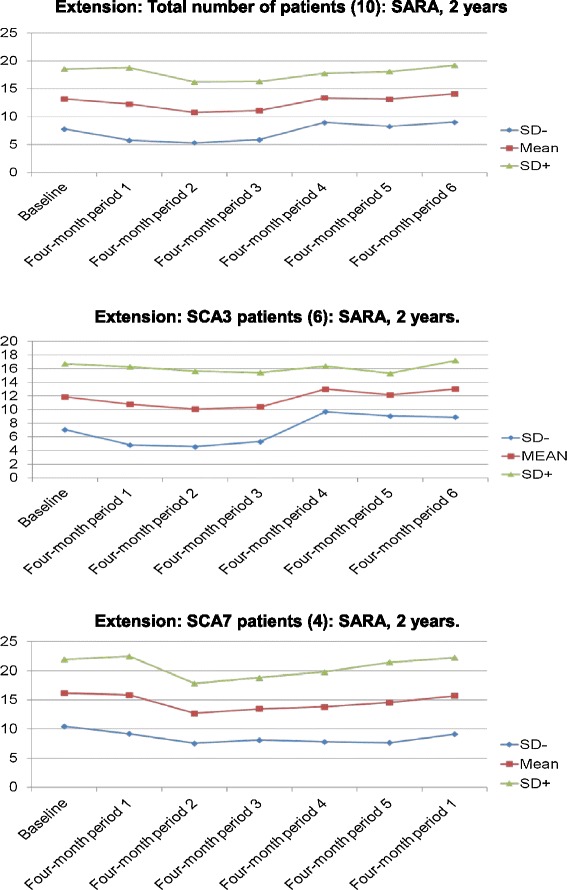
**These charts with mean and standard deviation lines show four-monthly score changes from baseline on the scale for the assessment and rating of ataxia (SARA) during two years of treatment with IGF-1.** Data from this extension study suggests that IGF-1 treatment might be effective for reducing the progression of SCAs long-term: Total number of patients (10) (top), SCA3 (6 patients) (middle), and SCA 7 (4 patients) (bottom) subgroups.

Data from our extension study suggests that IGF-1 treatment might be effective for reducing the progression of SCAs long-term (Figure 2; Table 2). These results must be interpreted under the limitations of the study design. Although they suggest that IGF-1 has a beneficial effect, full-fledged clinical trials are needed to prove that such an effect exists.

### SF-36v2

SF-36v2 scales showed that of the 26 patients, 18.5% were dissatisfied, 14.8% had poor satisfaction, 37% had fair satisfaction, and 29.6% showed high satisfaction during the limited study duration.

## Discussion

Exogenous trophic factors (such as glial derived neurotrophic factor and IGF-1) can delay the onset of hereditary Purkinje cell degeneration and gait ataxia in shaker mutant rats. These rats present spatially restricted degeneration of cerebellar Purkinje neurons from adult-onset heredodegeneration [11].

Serum levels of insulin, IGFs and IGF binding proteins (IGFBPs) are altered in human neurodegenerative diseases of various aetiologies, such as Alzheimer’s disease, amyotrophic lateral sclerosis, and cerebellar ataxia [27].

Two types of late-onset cerebellar ataxias (olivopontocerebellar and idiopathic cerebellar cortical atrophy) show low IGF-I levels in the blood but high levels of IGFBP-1 and IGFBP-3 [28].

Both ataxic animals and human patients with ataxia show altered serum IGF-1 levels. The pathological significance of this alteration, however, remains unknown. That said treatment with IGF-I has proven effective for neurotoxic and transgenic animal models of ataxia [29–31]. Mouse models of SCA1 and SCA7 showed a down-regulation of IGFBP-5 transcripts [15].

Serum levels of IGFs are also altered in two very different inherited neurodegenerative conditions, ataxia-telangiectasia and Charcot-Marie-Tooth 1A disease. Both types of patients have increased serum IGF-1 and IGFBP-2 levels, and decreased serum IGFBP-1 levels; while only ataxia-telangiectasia patients have high serum insulin levels [27]. Ataxia-telangiectasia and FRDA show cumulative DNA damage and might also show disturbed IGF-1 function [14]. DNA damage is known to reduce IGF-1 activity [32]. Altered serum levels of IGF-1 and IGFBPs have been reported in patients with late onset cerebellar ataxia [28].

IGF-1 normalised frataxin levels in frataxin-deficient neurons and astrocytes through the protein kinase B/mechanistic target of rapamycin signalling pathway. Furthermore, IGF-1 significantly increased levels of frataxin in cardiomyocites from conditional FRDA mouse mutants. These molecular improvements seen with IGF-1 also affect behaviour; for example, IGF-1 normalised motor coordination in the moderately FRDA-like transgenic mice [14].

IGF-1 has therapeutic effects for various types of cerebellar ataxias [13], exerting protective actions on mitochondrial function. Beneficial effects were observed with the use of IGF-1 therapy for FRDA patients. Participants in this proof-of-concept trial showed neurological improvement as measured by SARA and SF-36v2 scales. They also showed a decrease in neurological disease progression, together with possibly long-term stability of cardiac function. These data seem to indicate that IGF-1 therapy holds certain neurological and possibly cardiac benefits for patients with FRDA [Sanz-Gallego *et al.*, Cerebellum & Ataxias 2014, 1:10].

IGF-1 treatment has been tested in clinical trials for various disorders [13,33–36] and, with the exception of early clinical studies (that utilised very high doses of IGF-1 inducing transient hypoglycaemia), no significant adverse effects have been reported. In the present series, 1 patient with SCA7 was withdrawn because of consecutive fracture and fasting hypoglycaemia, 2 subjects presented common warts, 2 women gradually changed from straight hair to curly hair, and 1 woman showed an alteration in the frequency and duration of her menstrual cycle. In general, IGF-1 therapy was well tolerated by our patients with ADCA.

We have considered including only patients with SARA score between 8 and 28. So, the subject affection degree is perceived with clarity and, on the other hand, the patient can be assessed by means all items of the SARA scale (for example, gait and stance).

In the present study, we have included analysis and comparisons with previous data of Jacobi *et al.* work (26). This study provides a quantitative account of the natural history of four common SCAs (SCA1, SCA2, SCA3, and SCA6). It is based on an analysis of the first 2 years of the ongoing EUROSCA natural history study, a multicentric longitudinal cohort study of 526 patients. The advantages of this study are its prospective nature and the use of validated clinical scales (SARA). All patients had moderate disease severity. We consider that our patient’s characteristics resemble closely that cohort. The comparison of our results with those of Jacobi *et al.* in SCA3 and SCA6 [26], and our own SCA7 SARA score, indicated that our patients with ADCA who receive IGF-1 treatment showed a stabilization of disease progression. Data from our extension study suggests that IGF-1 treatment could be effective for reducing the progression of some SCAs long-term.

There exist limitations of this study. This was an open-label study, with a limited number of valid patients [11], a probable significant initial placebo effect, and a baseline score variability that influenced each individual’s evolution. We did not include placebo group and the number of extension study patients was limited to 10 due to budget restrictions.

## Conclusions

IGF-1 therapy was generally well tolerated by our patients with ADCA. The comparison of our data with that from a previous cohort indicates that IGF-1 treatment for patients with ADCA stabilizes disease development. This observation also suggests that IGF-1 therapy is able to decrease further progression of ADCA.

Further studies with more patients and double-blind placebo-controlled studies are necessary to more definitively assess the effectiveness of IGF-1 therapy. In the future, IGF-1 dosing might be changed from the conventional twice a day to once every 2 weeks by means of IGF-1 microsphere therapy [37].

## Methods

All participants provided written informed consent to participate in this open-label trial approved by the Institutional Ethical Committee of Clinical Research.

### Study design and end-points

We conducted a 1-year prospective open-label pilot clinical trial for patients with SCA3, SCA6, and SCA7 using 2-daily subcutaneous administrations of recombinant human IGF-1. A total of 27 out of the 30 initially recruited patients were considered valid for this study, and 26 patients completed it. Patients were recruited from the Unit of Hereditary Ataxia and Spastic Paraplegia at Hospital Universitario La Paz (Madrid, Spain). Subjects were required to have a SARA score between 8 and 28. Demographic and clinical variables of the 27 study patients are shown in Table 1. Baseline clinical characteristics were recorded including age, gender, height, weight, blood pressure, medical history and medications. Exclusion criteria were as follows: history of malignancy, active renal disease or other systemic illnesses that would make participation unsafe, diabetes mellitus or active major depression, the use of any investigational drugs within the previous 30 days, the inability to comply with requirements of the protocol; pregnancy, breastfeeding or planning to become pregnant during the study period.

The primary outcomes were safety concerns. The secondary outcome was the change at 4, 8 and 12 months in neurological symptoms measured with the scale for the assessment and rating of ataxia (SARA) [24] compared with the baseline for each type of SCA and SF-36v2 scores [25]. This change was also evaluated for the composite of all SCAs, as a single group.

Patients who consecutively completed this pilot clinical trial were presented the option to continue IGF-1 therapy in an open-label, single treatment, safety and efficacy, long-term extension study for a period of 1 year. A total of 12 patients, consecutively selected, were enrolled for this extension study, and 10 patients completed it.

### IGF-1 treatment

The intervention consisted of a subcutaneous injection of recombinant human IGF-1 (mecasermin, Increlex®; Ipsen-Pharma). A dose of 0.50 μg/kg body weight of IGF-1 was subcutaneously injected twice daily. This represented the highest tolerated dose in phase I human dose escalation studies, with hepatic toxicity being the dose limiting factor [38,39].

### Clinical evaluation

Safety assessments included:

1. Haematology assessments (consisting of haemoglobin, total and differential white blood cell count, absolute neutrophil count and platelet count) at baseline, every 4 months (±7 days) during the study, and at the end of the study or early termination, whichever came first.
2. Blood chemistry assessments (consisting of fasting glucose, alanine aminotransferase, aspartate aminotransferase, and creatinine) at baseline, every 4 months (±7 days), and at the end of the study or early termination, whichever came first.
3. Physical examination (including head, eyes, ears, nose, throat, cardiovascular, respiratory, musculoskeletal, dermatological, neurological, lymph nodes, endocrine/metabolic, gastrointestinal, genitourinary and reproductive examinations) at baseline, and at the end of the study or early termination, whichever came first.
4. Vital signs (weight, pulse, blood pressure and temperature) at baseline, every 4 months (±7 days) and at the end of the study, or early termination, whichever came first.
5. Electrocardiogram: at baseline, 12 months (±7 days) after start of the study, and at the end of the study or early termination, whichever came first.
6. Adverse events and medication(s): subjects were questioned about the occurrence of any adverse events and the use of any medication(s) at baseline, every 4 months (±7 days) during the study and at the end of the study (or early termination).
7. Urine pregnancy tests were performed at baseline, every 4 months (±7 days) and at the end of the study (or early termination) for all females of childbearing potential. Contraceptive counselling was also provided for all sexually active females.

### Efficacy analysis

Periodic functional analyses were conducted with SARA every 4 months (±7 days), and at the end of the study or early termination, whichever came first. Patient satisfaction with IGF-1 therapy was measured using the SF-36v2. This scale is a validated and widely used quality-of-life measurement originating from the Medical Outcomes Study. The survey consists of 36 multiple-choice health-related questions, grouped into eight multi-item domains measuring quality in different aspects of daily life (25). SF-36v2 is included in our Clinical Pathway of Degenerative Ataxias and Spastic Paraplegias.

### Statistical analysis

Safety analyses were performed on the safety intent-to-treat population, which was defined as selected patients who were administered at least one dose of the allocated drugs. Efficacy analyses were performed on the per protocol population, which was defined as those patients who had completed at least one 4-month period in the study and who had no major protocol violations.

For the efficacy assessments, the change from baseline to every 4 months and the end of the study was calculated. Non-parametric Wilcoxon-Mann–Whitney test were used to compare the change from baseline to post-baseline visits for all efficacy parameters. The evolution in time was studied (quarterly) using linear mixed-effects models for the adjustment of correlations caused by repeated measurements made on the same statistical units (longitudinal study). The aim was to determine the quarterly rate of change with a confidence interval (CI) of 95%. In 2011, a study performed on 139 patients with SCA3 reported that the mean SARA 1-year worsening of the cohort was 1.61 ± 0.12 (average ± SD) points. In this study, SARA progression in SCA6 (107 patients) was slowest and nonlinear (first year average ± SD: 0.35 ± 0.34, second year: 1.44 ± 0.34) [26]. These data were used for the comparison of our results. In case of SCA7 the annual worsening index matches our own patients’ annual worsening index prior to the beginning of the trial, with mean SARA 1-year worsening of the cohort was 1.5 ± 0.9 (average ± SD) points.
